# Intermolecular Forces Driving Hexamethylenetetramine Co-Crystal Formation, a DFT and XRD Analysis

**DOI:** 10.3390/molecules26195746

**Published:** 2021-09-22

**Authors:** Giovanni Bella, Francesco Nicolò, Giuseppe Bruno, Antonio Santoro

**Affiliations:** Department of Chemical, Biological, Pharmaceutical and Environmental Sciences, University of Messina, Viale F. Stagno d’Alcontres 31, 98166 Messina, Italy; giovanni.bella@unime.it (G.B.); fnicolo@unime.it (F.N.); giuseppe.bruno@unime.it (G.B.)

**Keywords:** hexamethylenetetramine, co-crystals, SCXRD, DFT analysis

## Abstract

Interest in co-crystals formation has been constantly growing since their discovery, almost a century ago. Such success is due to the ability to tune the physical-chemical properties of the components in solid state by avoiding a change in their molecular structure. The properties influenced by the co-crystals formation range from an improvement of mechanical features and chemical stability to different solubility. In the scientific research area, the pharmacological field is undoubtedly one of those in which an expansion of the co-crystal knowledge can offer wide benefits. In this work, we described the crystalline structure of hexamethylenetetramine co-crystallized with the isophthalic acid, and we compared it with another co-crystal, showing the same components but different stoichiometry. To give a wider overview on the nature of the interactions behind the observed crystal packing and to rationalize the reasons of its formation, a computational analysis on such structures was carried out.

## 1. Introduction

One of the main limits in the development of highly efficient drug molecules regards their low aqueous solubility with the consequent limitation of the bioavailability. There are several possible approaches to solve such a problem and this improves the water solubility and the dissolution rate. One of the oldest strategies regards the size reduction of the particles of the drug, which influence the kinetic of the dissolution rate. However, valid and modern approaches to enhance the solubility of the drug can be pursued in different ways, by combining the size reduction with different formulations of the drug. One of the most effective strategies for enhancing the solubility of acid/basic drugs consists of the formation of salts, using an appropriate partner for the drug [1]. More recently explored possibilities consist of co-crystallize the active drug with another suitable component [2], drug delivery by a suitable carrier [3,4] or resort to the formation of polymorphs. It is nowadays well known that the arrangement adopted by species in their solid form brings a change of their physical-chemical properties and can have profound effects on the dissolution rate [5,6], stability, and bioavailability of that species. Such properties and the possibility to predict them led to an increased economic interest by pharmaceutical companies on the study of the basic principles that can influence co-crystals, polymorphs and pseudo-polymorphs formation. The kinetic factors in a crystallization process are linked to the operating conditions. Some of them can be easily controlled, like temperature of crystallization, some others such as the presence of impurities can be hardly predicted. Among all the possible crystals, only the ones with sufficient kinetic stability are commercially and academically important. Unfortunately, it is impossible to take into account all of the kinetic factors that can generate and influence new crystal forms. The possible forms that a species can assume in its crystalline state could be predicted by taking in consideration the supramolecular aspects of the crystallization process. A supramolecular approach allows one to build complex structures with programmed properties [7,8] and shapes [9] in solution, through the correct understanding of all the thermodynamic and kinetic processes which govern the system. Such approach can be used in a solid state to study all the crystallization paths which can give different outputs such polymorphs, co-crystals or salts. It is important to consider how the molecules arranging themselves to build an energetically favored crystal packing and which kind of non-covalent forces are involved in the process. By taking advantage of computational chemistry, it is possible to evaluate the thermodynamic stability of many possible structures, and among them, select the most thermodynamically plausible ones.

In this general framework, we examine and attempt to provide some rationalization to a new co-crystal structure of a hexamethylenetetramine (HMTA) and isophthalic acid (IPA) containing a water molecule linked through hydrogen bonds to the crystal lattice. The HMTA is widely used in pharmacology to treat bladder and kidney bacterial infections. When pH = 5.5 in the urine, the HMTA undergoes a degradation process, converting into formaldehyde [10].

## 2. Results and Discussions

### 2.1. Structure Description

**Figure 1:**
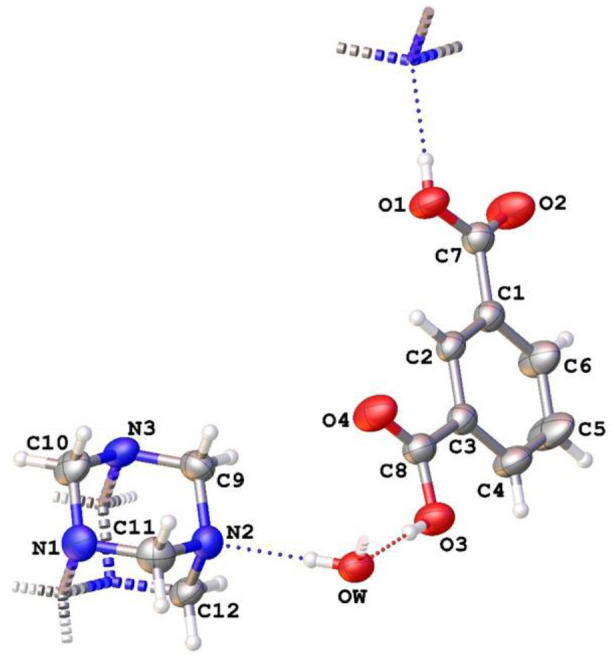
ORTEP diagram of the asymmetric unit of compound **1**. The hydrogen interactions are represented by dotted lines.

The crystallographic data for compound **1** collected at room temperature are presented in Table 1. The asymmetric unit (AU) of the supramolecular complex is constituted by half a molecule of water, IPA and HMTA. These components lie on two adjacent crystallographic planes {040}. While the acid molecule lies entirely on the 040 crystallographic plane, the HTMA is cut in two by the plane passing through the atoms N3C10C12N1 (see Figure 1).

The evidence of the co-crystal nature of the compound **1** is found in the bond distances of carboxylic fragments C=O (2.208 Å) and C-OH (1.322 Å), which clearly indicate no acid deprotonation.

**Figure 2:**
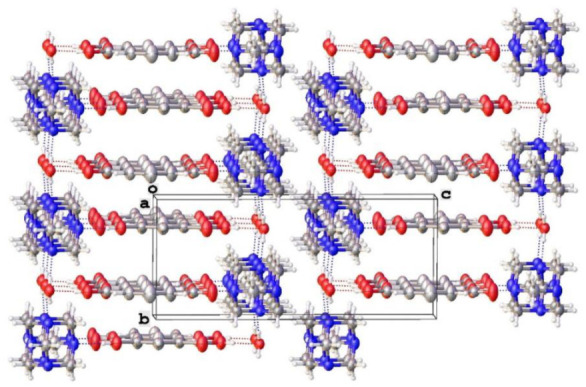
Molecular packing of compound **1**.

The whole molecular packing is mainly determined by an array of strong hydrogen bonds between all the components of compound **1,** with a non-negligible contribution of p-p stacking interactions at 3.452 Å, involving two centrosymmetric IPA molecules (as shown in Figure 2).

**Figure 3:**
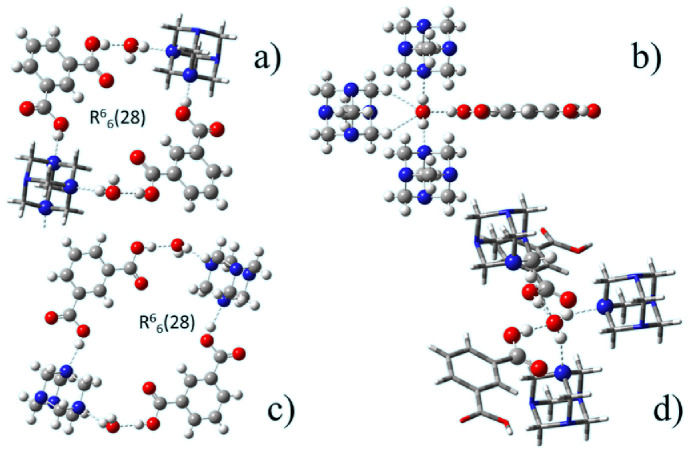
Hydrogen bond motif in compound **1** (**a,b**) and in compound **2** (**c,d**).

The water molecule is involved in three hydrogen bonds (Ow…O3 (2.643(2) Å), Ow…N2(N2′) (2.822(1) Å)), assisted by two weaker electrostatic interactions with methylenic hydrogens, as depicted in Figure 3b. In the same picture (Figure 3a), the graph set $R_{6}^{6}$(28) [11] motif generated by the HBs is illustrated. In the compound **1**, each molecule of IPA is directly bonded via HB to a N3 atom (N3…H-O1, 2.727(2) Å) and to the water molecule.

In the literature, some other structures constituted by the same molecular components of compound **1** (IPA, HMTA and water) are reported. These structures can differ from each other via molecular packing (polymorphs), the presence of one or more water molecules (pseudo-polymorph) and different stoichiometry. In particular, the compound **2** (MIPVIQ [12]) shown in Figure 3c–d exhibits the same $R_{6}^{6}$(28) interaction pattern of compound 1 (Figure 3a), showing a similar hydrogen network.

**Figure 4:**
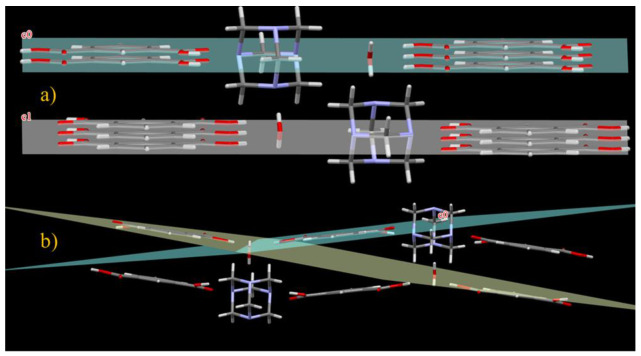
General packing in compound **1** (**a**) and in compound **2** (**b**).

Although the two co-crystals exhibit essentially the same intermolecular interactions, their whole molecular packing is largely different, as can be seen in Figure 4a,b, where their spatial distribution is compared.

The absence of conformational flexibility makes the hexamethylenetetramine a molecule which is structurally very stiff, however we observed that the Td symmetry is altered by the different HBs that the four nitrogen atoms can form; thus, indicative differences in the N-C bond distances can be observed. Nitrogen atom N1 not involved in any interaction shows the shortest N-C bonds distances (1.462 Å), while the others, N2-C and N3-C, are 1.474 Å and 1.477 Å, respectively. Although small, the observed differences in N-C bond distances are systematic and are attributed to the different strength of the intermolecular interactions involving the different nitrogen atoms of the HMTA.

In compound **1**, three nitrogen atoms of HTMA are involved in as many hydrogen bonds, the fourth nitrogen (N1) does not present any significant intermolecular interaction. While in the HTMA in compound **2**, all the four nitrogen atoms are involved in HB interactions, two of them with the OH group of the carboxylate (2.745 Å of bond distance) and the other two with water molecules (2.777 Å and 2.803 Å).

It is well known that HMTA usually participates in only two strong hydrogen bonds [13,14,15,16,17]. Only in cases where HTMA interacts with rich hydrogen donors, can it form three or four hydrogen bonds [18]. Such behaviour could be substantially attributed to the lower electron donor attitude of the HMTA when involved in multiple hydrogen bonds. This behaviour showed by the HMTA fragment could be a key feature to explain the abundance of co-crystal structures with HMTA in literature, in comparison to salt crystals. When the HMTA becomes protonated, its ability to form strong hydrogen bonds with the other non-protonated nitrogen atoms decreases. In the CSD [19] there are only three compounds in which the HMTA has two protonated nitrogen atoms and no tris or tetra protonated compounds are present [20,21,22].

In the synthesis of compound **1**, its co-crystal structure was predicted by using the ΔpKa rule, a general rule which help to predict crystal formation rather than salt. It is based on the relative values of pKa. In particular, when this difference (ΔpKa = pKa base–pKa acid) is greater than 2–3, the product obtained will be a salt [23,24]. The reliability of the prediction rules is important in several fields, with a particular focus in the pharmaceutical industry for the active pharmaceutical ingredients (APIs) with desirable properties. For complex **1**, the ΔpKa value corresponds to 1.67 (5.13 for HMTA [25], 3.36 for IPA [26]).

### 2.2. Computational Details

**Figure 5:**
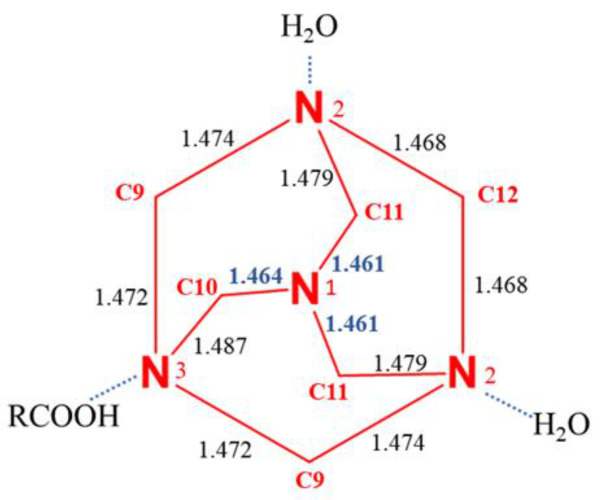
Schematic representation of the HTMA fragment in compound **1**, showing the bond distances in Angstrom.

**Figure 6:**
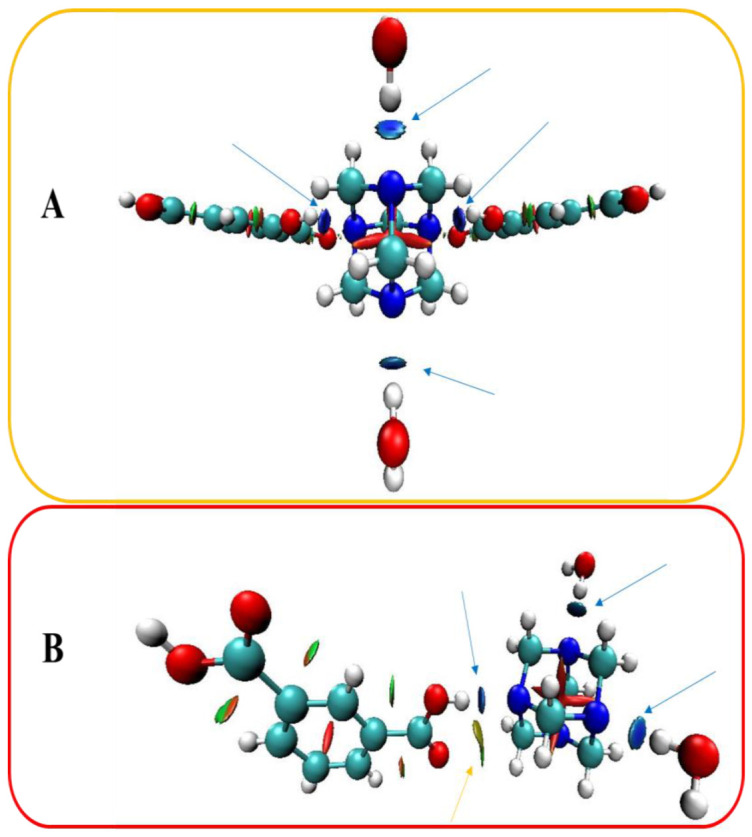
Non-covalent interaction surfaces regarding compound **2** (**A**) and compound **1** (**B**); blue arrows indicate the specific strong hydrogen bonds, while the green arrow highlights the intermolecular electrostatic interactions in the long-range regime.

The use of density functional theory (DFT) has become increasingly commonplace among chemists, providing them a useful tool for an accurate analysis of complex chemical species [27,28]. In this paper, single point energy was performed at CAM-B3LYP/6-311++G(d,p) level adding Grimme-dispersion terms (D3) [29] by means of Gaussian09 package [30]; a non-covalent interaction (NCI) was generated by Multiwfn code [31] with a high-quality grid and visualized using VMD [32]. Focusing on the crystal packing of compounds **1** and **2**, an accurate matching could be essential in order to visualize the difference between the two co-crystalline aggregates. In these terms, the occurrence of electrostatically favorable intermolecular interactions represents a useful indicator of energy stability of the crystal [33]. Figure 5 and Figure 6 clarifies how HMTA assumes a different electrostatic behavior in the two-crystal packing. In compound **2**, each nitrogen atom is involved in a strong hydrogen bond (blue pseudo circular surface) regarding two molecules of water and two IPA. As concerning compound **1**, only three nitrogen atoms establish hydrogen bonds (two water molecules and one IPA). In the latter, it is worth noting that a hydrogen bond between carboxylic scaffold and tertiary amine nitrogen is cooperatively assisted by an electrostatically favorable long-range interaction between carbonyl sp2-oxygen and hydrogens on methylene group (HMTA).

**Scheme 1:**
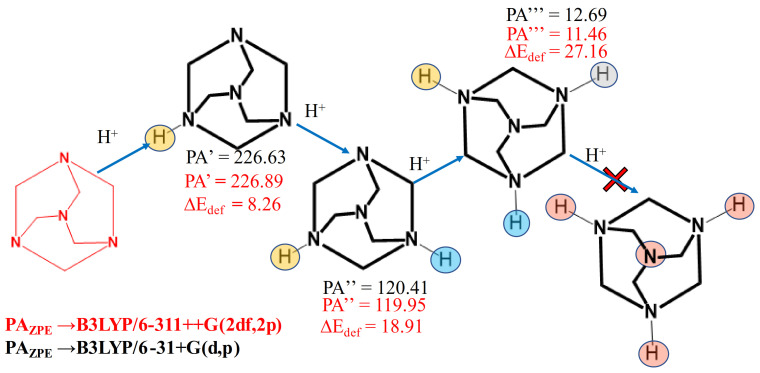
Energetic diagram of all the four step-by-step protonations of HMTA; for each step, the proton affinity (PA) and the deformation energy (ΔEdef) are displayed.

An important property that can be analysed by using computational methods is the proton affinity (PA). The proton affinity was considered as the negative of the protonation enthalpy (PA=-ΔH). The PA was computationally calculated at gas phase, at B3LYP/6-31+G(d,p) and B3LYP/ 6311++G(2df,2p) level. Scheme 1 shows the computed PA of mono, bis and tris-protonated forms. The values of the first three protonations, corrected for zero-point energy, together with the deformation energy, are reported in Scheme 1.

Deformation energy for each protonated species was computed as the difference between the ground state energy of previous neutral molecule or ions (HMTAnHn+) and the energy of the neutral molecule or ions with the same conformation of the optimized protonated species. As can be seen from the data shown in Scheme 1, the PA drastically decreases from the first to the third protonation. Although the calculated PA relative to the fourth protonation can be calculated, it is not reported, because its negative value has no chemical significance.

**Figure 7:**
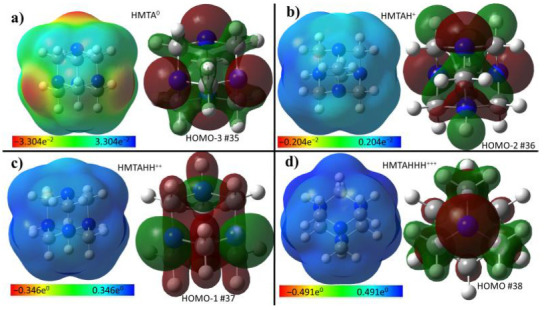
Electrostatic potential surface (ESP) and the molecular orbitals of HMTA in its neutral form (**a**), monoprotonated (**b**), bi protonated (**c**), and tri protonated (**d**).

Figure 7 illustrates the molecular electrostatic potential surface (ESP) and the MOs mainly centred on the available lone-pair of nitrogen atoms for the neutral HMTA and the three protonated species. The ESP pictures clearly spotlight the low attitude of HMTA to multiple protonations.

## 3. Materials and Methods

Chemicals and solvents used were purchased from commercial suppliers (Sigma Aldrich, Merk Life Science S.r.l., Milan, Italy) and were used without further purification.

The crystal was prepared by adding an ethanol solution of IPA (around 1 mmol) to a solution of HMTA (1 mmol). The reaction was stirred for a few minutes and then the solvent was left to evaporate at room temperature, to obtain, in a few days, colorless crystals.

A suitable crystal was selected and mounted on a Bruker APEX-II CCD diffractometer. The crystal was kept at 296.15 K during data collection. Using Olex2 [34], the structure was solved with the SHELXT [35] structure solution program using intrinsic phasing and refined with the SHELXL [36] refinement package using least squares minimization. A SCXRD analysis was made using several crystals of the same sample, obtaining, for all the data acquired, the same crystallographic cell. Here, we presented the result with the best R factor.

All the crystallographic data of compound **1** are reported in the Supplementary Materials.

## 4. Conclusions

In summary, we synthesized and studied, through single crystal X ray diffraction, a new co-crystal formed by HMTA, IPA and a water molecule. The obtained co-crystal obeys to the semi-empyrical rule of ΔpKa, showing a value of 1.67, within the expected range of 0–3. Supported by a DFT analysis, the relative abundance of similar co-crystals in CSD was attributed to the energetic unfavored HBs when HMTA is mono or bis protonated. A comparison based on SCXRD data with a species sharing the same components was performed in order to highlight the reasons for their different 3D arrangements. The major contribution to such differences turned out to be the number of hydrogen bonds which involve the HMTA fragment (three in compound **1** and four in compound **2**), whereas in compound **1**, the nitrogen not directly involved in a hydrogen bond exhibits two long range hydrogen interactions.

## Figures and Tables

**Table 1:** Crystal data and structure refinement for compound **1**.

Identification Code	Compound 1
Deposition number	2097042
Empirical formula	C_7_H_10_N_2_O_2.5_
Formula weight	162.17
Temperature/K	296.15
Crystal system	monoclinic
Space group	P2_1_/m
a/Å	6.8921(2)
b/Å	6.9032(2)
c/Å	16.5075(4)
α/°	90
β/°	101.9540(10)
γ/°	90
Volume/Å^3^	768.35(4)
Z	4
ρ_calc_ g/cm^3^	1.402
μ/mm^−1^	0.108
F(000)	344.0
Crystal size/mm^3^	0.48 × 0.35 × 0.18
Radiation	MoKα (λ = 0.71073)
2Θ range for data collection/°	6 to 54
Index ranges	−8 ≤ h ≤ 8, −8 ≤ k ≤ 8, −21 ≤ l ≤ 21
Reflections collected	27297
Independent reflections	1805 [R_int_ = 0.0272, R_sigma_ = 0.0109]
Data/restraints/parameters	1805/0/135
Goodness-of-fit on F^2^	1.035
Final R indexes [I>=2σ (I)]	R_1_ = 0.0349, wR_2_ = 0.0982
Final R indexes [all data]	R_1_ = 0.0391, wR_2_ = 0.1037
Largest diff. peak/hole/e Å^−3^	0.21/-0.16

## Data Availability

Not applicable.

## Supplementary Materials

The following are available online. Table S1: Fractional Atomic Coordinates (×10^4^) and Equivalent Isotropic Displacement Parameters (Å^2^ × 10^3^) for compound **1**. U_eq_ is defined as 1/3 of of the trace of the orthogonalised U_IJ_ tensor, Table S2: Anisotropic Displacement Parameters (Å^2^ × 10^3^) for compound **1**. The Anisotropic displacement factor exponent takes the form: −2π^2^[h^2^a*^2^U_11_+2hka*b*U_12_+…], Table S3: Bond Lengths for compound **1**, Table S4: Bond Angles for compound **1**, Table S5: Hydrogen Bonds for compound **1**, Table S6: Torsion Angles for compound **1**, Table S7: Hydrogen Atom Coordinates (Å × 10^4^) and Isotropic Displacement Parameters (Å^2^ × 10^3^) for compound **1**.

## Sample Availability

Not available.
