# Supplementary material for: Intermolecular Forces Driving Hexamethylenetetramine Co-Crystal Formation, a DFT and XRD Analysis
Source: Molecules. 2021 Sep 22;26(19):5746. doi: 10.3390/molecules26195746 (PMC8510214; doi:10.3390/molecules26195746)
Supplement: Supplementary file 1 [file molecules-26-05746-s001.zip › molecules-1324805-supplementary.pdf]

Intermolecular Forces Driving Hexamethylenetetramine Co-Crystal Formation, a DFT and XRD Analysis

Giovanni Bella, Francesco Nicolò, Giuseppe Bruno and Antonio Santoro *

Department of Chemical, Biological, Pharmaceutical and Environmental Sciences, University of Messina, Viale F. Stagno d'Alcontres 31, 98166 Messina, Italy; giovanni.bella@unime.it (G.B.); fnicolo@unime.it (F.N.); giuseppe.bruno@unime.it (G.B.)
* Correspondence: a-santoro@unime.it or antonio.santoro@unime.it

Table S1. Fractional Atomic Coordinates (×10^4) and Equivalent Isotropic Displacement Parameters (Å^2×10^3) for compound 1. U_{eq} is defined as 1/3 of of the trace of the orthogonalised U_{IJ} tensor.

Atom	*x*	*y*	*z*	U(eq)
C1	3938(2)	2500	-447.9(10)	35.9(3)
C2	5342(2)	2500	282.9(9)	33.8(3)
C3	4774(2)	2500	1043.3(9)	35.2(3)
C4	2773(3)	2500	1064.7(11)	49.5(4)
C5	1370(3)	2500	336.0(13)	69.4(7)
C6	1943(3)	2500	-418.3(12)	56.9(5)
C7	4513(2)	2500	-1269.8(10)	38.7(4)
O1	6438.5(17)	2500	-1224.8(7)	49.1(3)
O2	3320(2)	2500	-1916.7(8)	68.4(5)
C8	6352(2)	2500	1812.6(10)	39.7(4)
O3	5647.8(18)	2500	2505.4(7)	50.4(3)
O4	8105.1(18)	2500	1811.3(8)	60.5(4)
N1	14024.2(19)	7500	4235.2(8)	44.3(4)
N2	10963.7(13)	5734.0(14)	3695.8(6)	39.6(3)
N3	12606.6(19)	7500	2747.3(8)	42.0(3)
C9	11450.1(17)	5769.3(18)	2867.4(7)	43.2(3)
C10	14449(2)	7500	3401.4(11)	47.9(4)
C11	12838.3(18)	5788.5(19)	4322.8(7)	45.9(3)
C12	9842(2)	7500	3786.4(10)	39.9(4)
OW	8547.7(19)	2500	3833.6(7)	42.9(3)

Table S2. Anisotropic Displacement Parameters (Å^2×10^3) for compound 1. The Anisotropic displacement factor exponent takes the form: $-2\pi^2[h^2a^{*2}U_{11}+2hka^*b^*U_{12}+\ldots]$.

Atom	U_{11}	U_{22}	U_{33}	U_{23}	U_{13}	U_{12}
C1	33.0(7)	37.2(8)	38.1(8)	0	8.6(6)	0
C2	27.7(7)	35.3(7)	39.6(8)	0	9.5(6)	0
C3	33.7(7)	33.6(7)	38.9(8)	0	9.2(6)	0
C4	38.1(8)	71.1(12)	43.0(9)	0	17.1(7)	0
C5	27.3(8)	126(2)	56.5(11)	0	12.9(8)	0
C6	30.9(8)	94.2(15)	43.6(9)	0	3.5(7)	0
C7	35.4(8)	42.4(8)	38.0(8)	0	7.1(6)	0
O1	36.9(6)	75.6(9)	36.8(6)	0	11.9(5)	0

Table S2. Anisotropic Displacement Parameters (Å^2×10^3) for compound 1. The Anisotropic displacement factor exponent takes the form: -2π^2[h^{2}a*^{2}U$_{11}$+2hka*b*U$_{12}$+…].

Atom	U_{11}	U_{22}	U_{33}	U_{23}	U_{13}	U_{12}
O2	42.4(7)	124.5(14)	36.1(7)	0	2.9(5)	0
C8	38.4(8)	44.5(9)	37.4(8)	0	10.6(6)	0
O3	43.3(6)	72.6(9)	36.0(6)	0	9.8(5)	0
O4	35.5(6)	101.2(12)	44.1(7)	0	6.8(5)	0
N1	30.8(6)	61.0(9)	36.5(7)	0	-4.2(5)	0
N2	36.9(5)	43.5(5)	37.2(5)	0.5(4)	5.2(4)	-4.1(4)
N3	31.0(6)	63.8(9)	31.9(6)	0	7.8(5)	0
C9	39.7(6)	52.4(7)	36.4(6)	-9.6(5)	5.3(4)	-0.7(5)
C10	25.3(7)	70.2(12)	47.6(9)	0	5.8(6)	0
C11	45.0(6)	50.9(7)	38.0(6)	7.3(5)	0.1(5)	7.0(5)
C12	28.8(7)	55.7(10)	36.3(8)	0	9.5(6)	0
OW	45.4(7)	47.8(7)	37.8(6)	0	13.8(5)	0

Table S3. Bond Lengths for compound 1.

Atom	Atom	Length/Å	Atom	Atom	Length/Å
C1	C2	1.381(2)	C8	O4	1.209(2)
C1	C6	1.386(2)	N1	C10	1.465(2)
C1	C7	1.490(2)	N1	C11[1]	1.4608(15)
C2	C3	1.390(2)	N1	C11	1.4608(15)
C3	C4	1.387(2)	N2	C9	1.4740(14)
C3	C8	1.491(2)	N2	C11	1.4792(14)
C4	C5	1.378(3)	N2	C12	1.4679(13)
C5	C6	1.382(3)	N3	C9	1.4723(14)
C7	O1	1.3137(19)	N3	C9[1]	1.4723(14)
C7	O2	1.205(2)	N3	C10	1.487(2)
C8	O3	1.3316(19)			

[1]+X,3/2-Y,+Z

Table S4. Bond Angles for compound 1

Atom	Atom	Atom	Angle/°	Atom	Atom	Atom	Angle/°
C2	C1	C6	119.32(15)	O4	C8	O3	122.95(15)
C2	C1	C7	121.65(13)	C11[1]	N1	C10	108.52(8)
C6	C1	C7	119.02(15)	C11	N1	C10	108.52(8)
C1	C2	C3	120.77(14)	C11	N1	C11[1]	107.96(13)
C2	C3	C8	118.51(13)	C9	N2	C11	108.38(9)
C4	C3	C2	119.36(15)	C12	N2	C9	107.86(10)
C4	C3	C8	122.13(14)	C12	N2	C11	108.05(10)
C5	C4	C3	119.94(16)	C9	N3	C9[1]	108.49(12)
C4	C5	C6	120.46(16)	C9	N3	C10	107.87(8)
C5	C6	C1	120.15(16)	C9[1]	N3	C10	107.87(8)
O1	C7	C1	113.87(13)	N3	C9	N2	111.83(9)
O2	C7	C1	123.04(15)	N1	C10	N3	112.07(12)
O2	C7	O1	123.09(15)	N1	C11	N2	112.14(9)
O3	C8	C3	113.60(13)	N2[1]	C12	N2	112.30(12)

Table S4. Bond Angles for compound 1

Atom	Atom	Atom	Angle/°	Atom	Atom	Atom	Angle/°
O4	C8	C3	123.45(15)				

[1]+X,3/2-Y,+Z

Table S5. Hydrogen Bonds for compound 1.

D	H	A	d(D-H)/Å	d(H-A)/Å	d(D-A)/Å	D-H-A/°
O1	H1	N3[1]	0.82	1.91	2.7267(18)	175.8
O3	H3	OW	0.82	1.82	2.6432(17)	177.2
OW	HW	N2	0.847(17)	1.975(17)	2.8223(12)	179.0(17)

[1]2-X,1-Y,-Z

Table S6. Torsion Angles for compound 1

A	B	C	D	Angle/°	A	B	C	D	Angle/°
C1	C2	C3	C4	0.000(0)	C7	C1	C6	C5	180.000(0)
C1	C2	C3	C8	180.000(0)	C8	C3	C4	C5	180.000(0)
C2	C1	C6	C5	0.000(0)	C9	N2	C11	N1	58.19(13)
C2	C1	C7	O1	0.000(0)	C9	N2	C12	N2[1]	-59.24(14)
C2	C1	C7	O2	180.000(0)	C9[1]	N3	C9	N2	-58.27(14)
C2	C3	C4	C5	0.000(0)	C9	N3	C10	N1	-58.50(7)
C2	C3	C8	O3	180.000(0)	C9[1]	N3	C10	N1	58.50(7)
C2	C3	C8	O4	0.000(0)	C10	N1	C11	N2	-58.29(13)
C3	C4	C5	C6	0.000(0)	C10	N3	C9	N2	58.33(12)
C4	C3	C8	O3	0.000(0)	C11[1]	N1	C10	N3	-58.54(8)
C4	C3	C8	O4	180.000(0)	C11	N1	C10	N3	58.54(8)
C4	C5	C6	C1	0.000(0)	C11[1]	N1	C11	N2	59.15(15)
C6	C1	C2	C3	0.0	C11	N2	C9	N3	-58.34(12)
C6	C1	C7	O1	180.0	C11	N2	C12	N2[1]	57.72(14)
C6	C1	C7	O2	0.000(0)	C12	N2	C9	N3	58.41(12)
C7	C1	C2	C3	180.000(0)	C12	N2	C11	N1	-58.44(13)

[1]+X,3/2-Y,+Z

Table S7. Hydrogen Atom Coordinates (Å×10^4) and Isotropic Displacement Parameters (Å^2×10^3) for compound 1.

Atom	*x*	*y*	*z*	U(eq)
H2	6682.04	2500	265.98	41
H4	2377.87	2500	1570.29	59
H5	29.59	2500	352.02	83
H6	987.25	2500	-907.14	68
H1	6664.15	2500	-1693.37	74
H3	6574.44	2500	2907.21	76
H9A	12204.43	4618.21	2796.08	52
H9B	10231.98	5747.7	2449.63	52
H10A	15229.65	6363.96	3335.46	58
H10B	15229.65	8636.04	3335.46	58

Table S7. Hydrogen Atom Coordinates (Å×10^4) and Isotropic Displacement Parameters (Å^2×10^3) for compound 1.

Atom	*x*	*y*	*z*	U(eq)
H11A	12537.46	5781.6	4871.43	55
H11B	13605.04	4634.66	4269.13	55
H12A	8617.2	7500.02	3371.92	48
H12B	9498.13	7499.98	4327.28	48
HW	9280(20)	3470(30)	3798(10)	67(5)

1. Dolomanov, O.V., Bourhis, L.J., Gildea, R.J, Howard, J.A.K. & Puschmann, H. (2009), J. Appl. Cryst. 42, 339-341.
2. Sheldrick, G.M. (2015). Acta Cryst. A71, 3-8.
3. Sheldrick, G.M. (2015). Acta Cryst. C71, 3-8.
